# Loss of PLK2 induces acquired resistance to temozolomide in GBM via activation of notch signaling

**DOI:** 10.1186/s13046-020-01750-4

**Published:** 2020-11-11

**Authors:** Wahafu Alafate, Dongze Xu, Wei Wu, Jianyang Xiang, Xudong Ma, Wanfu Xie, Xiaobin Bai, Maode Wang, Jia Wang

**Affiliations:** 1 Department of Neurosurgery, The First Affiliated Hospital of Xi’an Jiaotong University, 277 Yanta West Road, Xi’an, Shaanxi 710061 P.R. China; 2 Center of Brain Science, The First Affiliated Hospital of Xi’an Jiaotong University, 277 Yanta West Road, Xi’an, 710061 Shaanxi China

**Keywords:** GBM, PLK2, Notch signaling, TMZ, Chemoresistance

## Abstract

**Background:**

Glioblastoma (GBM) is a lethal type of primary brain tumor with a median survival less than 15 months. Despite the recent improvements of comprehensive strategies, the outcomes for GBM patients remain dismal. Accumulating evidence indicates that rapid acquired chemoresistance is the major cause of GBM recurrence thus leads to worse clinical outcomes. Therefore, developing novel biomarkers and therapeutic targets for chemoresistant GBM is crucial for long-term cures.

**Methods:**

Transcriptomic profiles of glioblastoma were downloaded from gene expression omnibus (GEO) and TCGA database. Differentially expressed genes were analyzed and candidate gene PLK2 was selected for subsequent validation. Clinical samples and corresponding data were collected from our center and measured using immunohistochemistry analysis. Lentiviral transduction and in vivo xenograft transplantation were used to validate the bioinformatic findings. GSEA analyses were conducted to identify potential signaling pathways related to PLK2 expression and further confirmed by in vitro mechanistic assays.

**Results:**

In this study, we identified PLK2 as an extremely suppressed kinase-encoding gene in GBM samples, particularly in therapy resistant GBM. Additionally, reduced PLK2 expression implied poor prognosis and TMZ resistance in GBM patients. Functionally, up-regulated PLK2 attenuated cell proliferation, migration, invasion, and tumorigenesis of GBM cells. Besides, exogenous overexpression of PLK2 reduced acquired TMZ resistance of GBM cells. Furthermore, bioinformatics analysis indicated that PLK2 was negatively correlated with Notch signaling pathway in GBM. Mechanically, loss of PLK2 activated Notch pathway through negative transcriptional regulation of HES1 and degradation of Notch1.

**Conclusion:**

Loss of PLK2 enhances aggressive biological behavior of GBM through activation of Notch signaling, indicating that PLK2 could be a prognostic biomarker and potential therapeutic target for chemoresistant GBM.

## Background

Glioblastoma (GBM) is one of the most aggressive tumors in central nervous system [1] with a median overall survival less than 15 months due to the very nature of GBM [2]. The current standard strategy of GBM including maximum safe resection, radiotherapy and the temozolomide (TMZ) chemotherapy are widely applied, however, most GBM patients still develop rapid tumor recurrence within a period of 7 months [3]. Accumulating evidence indicates that acquired chemoresistance to TMZ is the primary cause of therapy failure thus leads to eventual tumor recurrence and unfavorable prognosis [4]. Therefore, identifying reliable prognostic biomarker and potential therapeutic targets becomes a major obstacle in GBM treatment.

Treatments targeting kinases have taken researchers into a new insight of anti-tumor strategies, by which the proliferation of multiple cancers are significantly eliminated [5–7]. A wide range of kinases have been identified to be functionally required for tumorigenesis and therapy resistance in GBM [8]. Besides these kinases which function as oncogenes, there are also a portion of tumor-suppressing kinases promotes aggressive biological behaviors of GBM via eliminated expression or inactivation. Serine/threonine-protein kinase polo like kinase 2 (PLK2) is a key regulator participates in centriole duplication [9], G1/S phase transition [10] and synaptic plasticity [11]. Benetatos et al. [12] report that PLK2 expression is significantly suppressed in acute myeloid leukemias. It has been proved that CpG island methylation at the 5′- of promoter area induces ectopic expression of PLK2 and promote apoptosis in B-cell malignancies [13]. Additionally, in acute myeloma cell lines, targeting PLK2 by miR-126 induces the down-regulation of PLK2 and inhibits cell apoptosis and increases cell viability [14]. Moreover, the same microRNA was closely associated with TMZ resistance according to NoncoRNA database [15]. However, the potential function of PLK2 in GBM carcinogenesis and chemoresistance remains unclear.

Notch signaling pathway is one of the most conservative pathways that regulates essential processes during cell proliferation, migration, stem cell maintenance, apoptosis and differentiation [16, 17]. In mammals, there are 4 homologous proteins named Notch1, Notch2, Notch3 and Notch4, function as cytoplasmic receptors by binding to 2 ligands families, including Delta-like (DLL1–4) and Jagged (JAG1–2) [18, 19]. The interaction between Notch and its ligands triggers a conformational change in the Notch receptor and releases the active form of Notch Intracellular Domain (NICD) into the cytoplasm, which allows the transcription of Notch target genes, including HES1, c-MYC and cyclin D3 [20, 21]. Previous studies have reported the overexpression of Notch components including DLL1, Notch1 and ASCL1 correlates with a higher grade of glioma and a worse prognosis, indicating an activated Notch signaling is usually associated with more aggressive tumor phenotype [22–24]. Moreover, the overexpression of Notch1 promotes AKT activation, leads to the nuclear localization of NF-κB and β-catenin thus promotes cell migration and invasion [25]. Additionally, the elevation of several Notch-related genes are found under hypoxic condition and closely associated with poor outcome, indicating that Notch-related genes might hold a prognostic implication [18]. Interestingly, Purow et al. showed that Notch1 transcriptionally regulates EGFR, which is proved to be highly increased in GBM, through TP53 [26], Consistently, transcription of Notch signaling mediator genes are significantly overexpressed in the molecular subset of GBM with EGFR amplification [27]. As Notch signaling is essential for multiple malignancies of GBM, it is crucial to investigate the underlying molecular mechanisms of Notch pathway in GBM.

In this study, we found PLK2 is down-regulated in GBM and the elevation of PLK2 is positively correlated with chemosensitivity and favorable prognosis in GBM patients. Additionally, overexpression of PLK2 attenuated the proliferation and enhanced chemosensitivity of GBM cells. Moreover, we demonstrated that loss of PLK2 induced chemoresistance to TMZ by activation of Notch signaling via transcriptional regulation of HES1 and Notch degradation. Altogether, PLK2-Notch axis could be a prognostical biomarker and potential therapeutic target for TMZ resistant GBM.

## Methods and materials

### Differential gene expression analysis

Expression profile of GSE68029 [28] and GSE16011 [29] were extracted from Gene expression omnibus (GEO). These transcriptome profiles were preprocessed as previously described [30]. Gene expression datasets of TCGA GBM was extracted from GDC Data Portal (https://portal.gdc.cancer.gov/). The limma package [31] was used for identifying the differentially expressed genes in these datasets. The expression difference of individual gene was defined by log_2_(Fold change) and adjusted *P* value, in which log_2_FC < − 1 with an adjusted *P* value < 0.05 was defined as a down-regulated gene. Venn diagram was carried out to illustrate the overlapping down-regulated genes in these datasets.

### Ethical statement and patient samples

This study was approved by the Scientific Ethics Committee at the First Affiliated Hospital of Xi’an Jiaotong University (approval no. 2016–18). All the patients’ samples used in this study have obtained necessary consent, and all samples were embedded in paraffin blocks as previously described [30].

### Immunohistochemistry (IHC) and global immunohistochemistry score (GIS)

Immunohistochemistry was performed as previously described [32, 33]. Anti-PLK2 primary antibodies were purchased from Abcam (cat. no. ab71311). Secondary antibodies including Goat anti-rabbit IgG (cat. no. ab97051, Abcam) and goat anti-mouse IgG (cat. no. ab205719, Abcam) were used in this study. Tissues embedded with paraffin were cut into 4-mm sections followed by deparaffinized, rehydrated, and stained with primary antibodies at 4 °C overnight. The slides were then incubated with secondary antibodies and stained with DAB. Consequently, the slides were counterstained with hematoxylin and images were taken under the light microscope.

The global immunohistochemistry score (GIS) was used to measure the relative expression of PLK2 in different samples. GIS was calculated by the following equation: immunoreactivity score = staining intensity score × positive cell score. The staining intensity score was scored as: 0, negative; 1, weakly positive; 2, moderately positive; and 3, strongly positive. The positive cell score was scored as: 0, negative; 1, < 10% positive; 2, 11–50% positive; 3, 51–80% positive; and 4, > 80% positive. Consequently, an immunoreactivity score > 5 was considered as high expression, and ≤ 5 was defined as low expression.

### Lentivirus production and transduction

Lentivirus production and transduction were performed as described previously [30]. The lentiviral overexpression/knockdown PLK2 was designed and synthesized by Genechem (Shanghai, China). These lentiviruses were introduced into U87 and U251 cell lines according to manufacturer’s instruction. Stable clones transduced with PLK2 and shPLK2 were selected for 4 weeks by puromycin. Lentiviral particles packaging the shRNA are targeting PLK2 (5′-TAGTCAAGTGACGGTGCTG-3′) and the scramble control (5′-TTCTCCGAACGTGTCACGT-3′).

### Cell culture, in vitro cell proliferation and cell viability assay

GBM cell lines including U87 and U251 were purchased from Cell bank, Type culture collection, Chinese Academy of Sciences (Xi’an China) as previously described [30]. Cells were cultured in DMEM-F12 containing 10% FBS and antibiotics (1% penicillin and streptomycin). U87 and U251 cells were cultured in a humidified condition containing 5% CO_2_ at 37 °C.

For in vitro cell proliferation assay, cells were suspended adequately before seeded into 96-well plates at a density of 1 × 10^3^ cells/well. Cell number was counted by using alamarBlue following the manufacturer’s instruction after indicated treatments.

Cell viability assays were conducted as previously described [30]. In brief, cell number was calculated by cell counter with trypan blue, cells were then seeded into 96-cell plates after suspended adequately at a density of 2 × 10^3^ cells/100 uL per well. After 24 h of culturing, U87 and U251 cell lines received indicated in vitro chemotherapy using corresponding concentration of TMZ. At last, cell number was counted by alamarBlue and IC50 was calculated by SPSS 22.0.

### Quantitative RT-PCR (qRT-PCR) and Western blot analysis

The Quantitative RT-PCR assays were conducted as previously described [30]. In brief, total RNA was extracted using RNeasy mini kits according to the manufacturer’s instruction, and the concentration of RNA was determined by Nanodrop 2000. qRT-PCR was performed following the synthesis of cDNA according to the standard protocols. GAPDH was used as an internal control. Relative mRNA expressions were calculated by 2^-ΔΔt^ method. The primer sequences were listed as below:

PLK2: forward, 5′-GCTGATGTCTGGCTGTTCATCAG-3′ and reverse, 5′-CTTCCCTGTAGATCTCACA GTG-3′;

HES1: forward, 5′-AGTGAAGCACCTCCGGAAC-3′ and reverse, 5′-TCACCTCGTTCATGCACTC-3′.

c-MYC: forward, 5′- AAACACAAACTTGAACAGCTAC-3′ and reverse, 5′- ATTTGAGGCAGTTTACATTATGG-3′.

p21: forward, 5′- GGCATAGAAGAGGCTGGTGG-3′ and reverse, 5′- ATGGCGCCTGAACAGAAGAA-3′.

Cyclin D3: forward, 5′- AAACTTGGCTGAGCAGAGCA-3′ and reverse, 5′- GAACAGAGCCAGTCTCCACC-3′.

GAPDH: forward, 5′-ACCCAGAAGACTGTGGATGG-3′ and reverse, 5′-TTCAGC TCAGGGATGACCTT-3′.

Western blot analyses were performed as previously described [32]. Antibodies used in this study were shown as below: Anti-PLK2 primary antibody was purchased from Abcam (cat. no. ab71311). Anti-β-actin antibody was purchased from Abcam (cat. no. ab115777). Anti-NOTCH1, anti-NOTCH2, anti-HES1, anti-c-MYC and anti-cyclin D3 primary antibodies were bought from Cell Signaling Technology (CST) with the catalog number of #4147, #5732, #11988, #18583 and #2936, respectively. Additionally, Anti-Rabbit IgG and Anti-Mouse-IgG were purchased from CST (cat. no. #7074 and #7076, respectively).

### Colony formation assay and wound healing assay

Colony formation assays were conducted to detect the tumorigenic potential of U87 and U251 cell lines. Different numbers of stable U87 and U251 cells transduced with lentiviral PLK2 or vector were seeded into 6-well plates under indicating conditions. After cultured for 14 days to form colonies, these cells were fixed with methanol and stained with methylene blue.

The wound healing assay is used to study the migratory ability of U87 and U251 GBM cell lines. Cells were seeded in 6-well plates after adequately suspended. Afterwards, a sterile pipette tip was used to produce a wound line when cells reached the appropriate confluence. These cells were then washed at least 3 times to be cultured in FBS-free medium. Images were taken under inverted microscope at the same location. The leading edges were marked by white lines, and the relative distance of the borders was measure by Image J software.

### The construction of TMZ resistant glioma cell lines

To construct TMZ resistant cell lines, U87 and U251 were gradually treated with increasing concentration of TMZ. In brief, the beginning concentration of TMZ was set according to the IC50 of U87 and U251 naïve cells. The medium was changed every 2 days to keep the concentration of TMZ at a stable level for 2 weeks. Afterwards, new IC50 was calculated using the pre-treated cell lines and the process was continued until the IC50 of TMZ reached the amount of 500uM. These cells were then used for subsequent experiments.

### In vivo intracranial xenograft tumor models

The usage of experimental animals in this study was approved by the Ethics Committee of the School of Medicine, Xi’an Jiaotong University (approval no. 2016–085). In vivo xenograft model was constructed by using 6-week-old female nude mice. U87 and U251 cells transduced with indicating lentiviruses was suspended and diluted to the density of 1 × 10^5^ cells in 2 uL PBS then slowly injected into the nude mice brains. Each group of treatment contains five mice, and they were monitored until the following symptoms were observed: unsteady gait, arched back, more than 10% weight loss or leg paralysis.

### Flow cytometry

Flow cytometry assays were performed as previously described [30]. U87 TMZ resistant cells were transfected with either empty vector or plk2 overexpression lentivirus then treated with 200uM TMZ for 24 h. DMSO were used as a negative control. The Alexa Fluor® 488 Annexin V/Dead Cell Apoptosis kit was used strictly following the manufacturer’s protocol to measure the cell apoptotic rate.

### Gene set enrichment analysis (GSEA)

The transcriptome profiles of previously mentioned datasets were read into R programming and preprocessed by background correction, gene ID transformation and normalization. These data were then ordered by the expression level of PLK2 to divide all samples into two groups (PLK2^high^ and PLK2^low^) by quartile cutoff. Afterwards, these profiles were uploaded into the GSEA software [34] strictly following the guideline of the software to elucidate the enriched KEGG pathways that are significantly enriched in PLK2^low^ groups with the number of permutations set at 1000.

### Glutathione S-transferase (GST) pull-down assay

The ubiquitination level of Notch1 was determined by GST-mediated pull-down assays (Thermo Scientific, Rockford, IL). Briefly, GST-tagged Notch1 was transfected into indicated cell lines (isogenic PLK2 overexpressed cells or their negative control) by lipofectamine according to manufacturer’s instruction. After 24 h, cell lysates were collected and incubated with GST beads for 1 h. Then, GST complexes were washed extensively with lysate buffer and used to perform western blot assays.

### Statistical analysis

All the results in this study are exhibited as mean ± SD (Standard deviation) as described previously [35]. The number of independent replications is presented in the figure legends. Two-tailed *t* tests were conducted to evaluate statistical differences and One-way ANOVA following Dunnett’s post-test was used to compare the statistics in more than two groups. Kaplan-Meier survival analyses were performed using log-rank test. In addition, all statistical analysis was performed by GraphPad Prism 7 or SPSS 22.0 software, and statistical significance was defined as a two-sided *P* value less than .05 unless specifically indicated.

## Results

### PLK2 is significantly down-regulated in TMZ resistant GBM

**Fig. 1:**
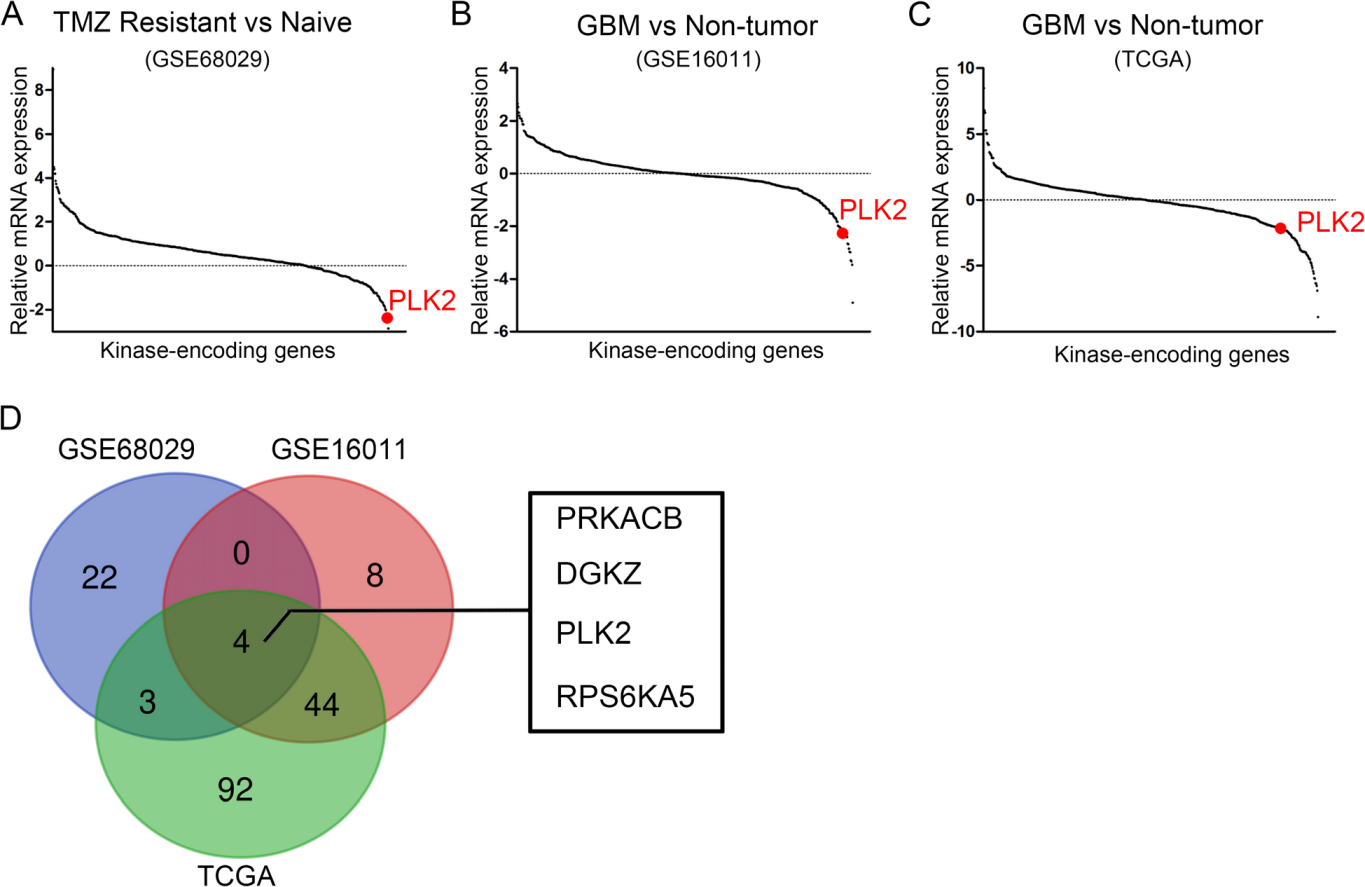
PLK2 is significantly down-regulated in TMZ resistant GBM. **a**, Microarray analysis for differentially expressed kinases (DEKs) in TMZ resistant cells versus Naïve cells using GEO database (GSE68029). **b**, Microarray analysis for DEKs in GBM versus non-tumor tissues in GEO database (GSE16011). **c**, Differential kinase expression analysis in GBM versus non-tumor tissues using TCGA GBM dataset. **d**, Venn diagram for down-regulated kinases in both three datasets of comparisons, indicating PLK2 was one of the most significantly down-regulated kinases in TMZ resistant cells as well as GBM tissues

As well known, aberrant expression and activation of kinases result in oncogenesis of a wide range of cancers including GBM [36]. Therefore, to investigate the differentially expressed kinase-encoding genes in TMZ resistant GBMs, hierarchical bi-clustering was performed by using previously published GEO database (GSE68029) [28]. The differentially expressed genes were then aligned with the 668-known kinase-encoding gene symbols. As is shown in Fig. 1a and Supplementary Table 1, PLK2 was among the most significantly suppressed kinase-encoding genes in TMZ resistant group. In addition, the differentially expressed genes of GBM versus non-tumor tissue were analyzed by using GSE16011 [29] and TCGA datasets. The results showed that PLK2 was consistently down-regulated in these 2 databases (Fig. 1b, c; Supplementary Table 2, 3). As is illustrated in Fig. 1d, Venn diagram was drawn to find out the overlapping down-regulated genes. Totally, 4 genes were negatively correlated with both TMZ resistance and GBM tissues, including PRKACB, DGKZ, PLK2 and RPS6KA5. Taken together, PLK2 showed significantly differential expression in correlation with GBM and TMZ resistance. Therefore, we picked PLK2 as a candidate gene in this study.

### Decreased PLK2 expression is closely associated with poor outcomes in glioma patients

**Fig. 2:**
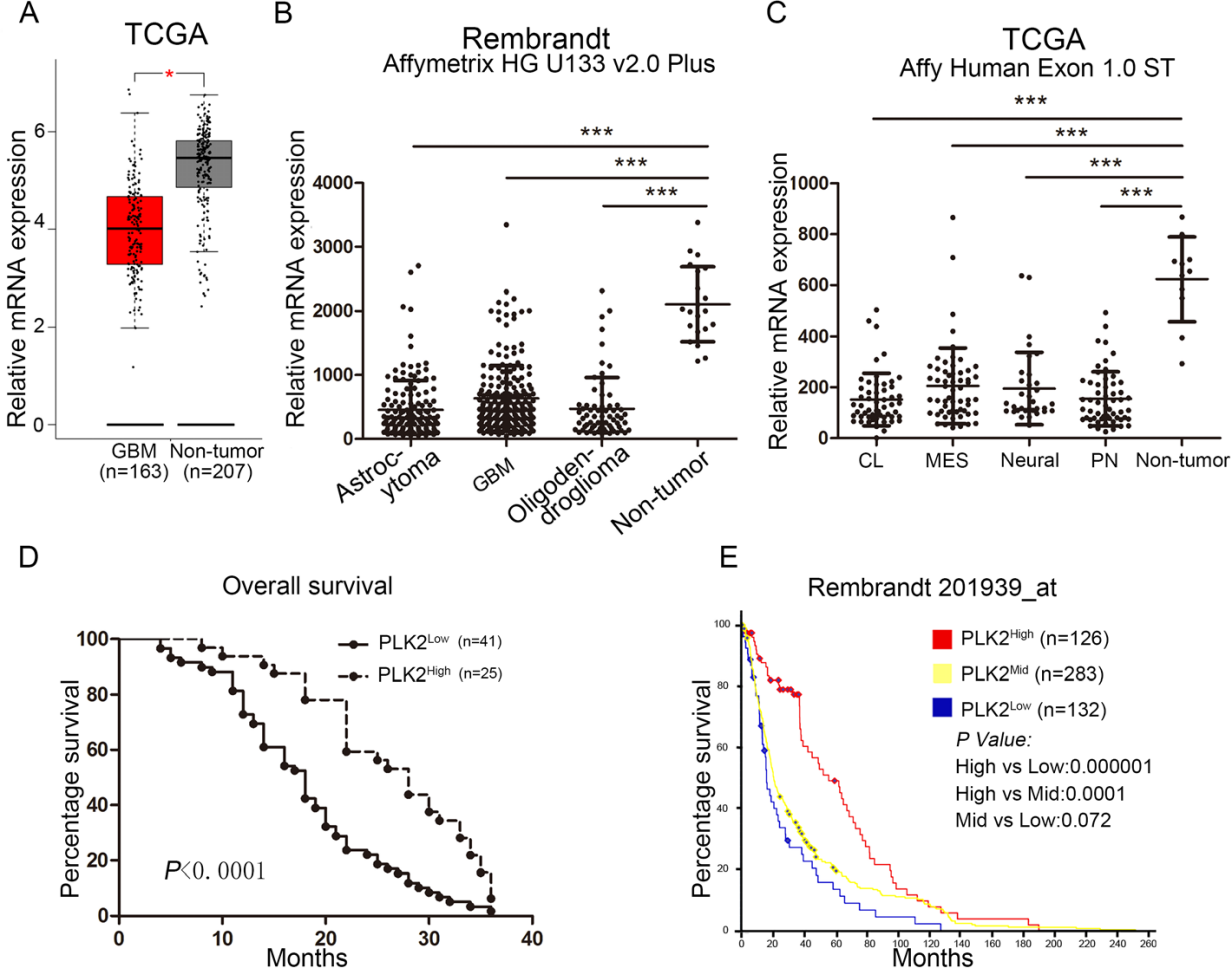
Decreased PLK2 expression is closely associated with poor outcomes in glioma patients. **a**, Gene expression analysis using TCGA GBM database (*P < .05, with student’s t-test). **b**, Gene expression analysis with Rembrandt database in different types of glioma versus non-tumor (*P < .05, **P < .01, ***P < .001, with one-way ANOVA followed by Dunnett’s posttest). **c**, Gene expression analysis with TCGA GBM dataset in different subtypes of GBM versus non-tumor (***P < .001, with one-way ANOVA followed by Dunnett’s posttest). **d**. Kaplan-Meier analysis for PLK2 expression using GBM patient samples (*P* < .0001, with log-rank test). **e**, Kaplan-Meier analysis for PLK2 expression in Rembrandt dataset by dividing glioma samples into 3 groups including PLK2^high^, PLK2^mid^, PLK2^low^ (with log-rank test within each comparison)

To gain more insight into the clinical relevance of PLK2 expression in GBM, TCGA GBM database were analyzed by using the online server GEPIA (http://gepia.cancer-pku.cn/) [37]. As is shown in Fig. 2a, PLK2 was significantly down-regulated in GBM compared with non-tumor tissues. Furthermore, Rembrandt database was used to investigate the expression pattern of PLK2 in different pathological subtypes of glioma including astrocytoma, GBM and oligodendroglioma. It is obvious that non-tumor tissues exhibited the highest expression of PLK2 compared with other types of glioma (Fig. 2b). Moreover, expression of PLK2 were found to be remarkably suppressed in all 4 molecular subtypes of GBM including classical (CL), mesenchymal (MES), neural and proneural (PN) when compared to non-tumor tissues (Fig. 2c, Supplementary Fig. 1C). These data suggested that loss of PLK2 was a significant biomarker for glioma/GBM, which lead us to investigate more deeply into the clinical relevance of PLK2 in GBM. Herein, IHC staining was performed to evaluate the expression of PLK2 in samples derived from patients underwent surgical resection in the Department of Neurosurgery, The First Affiliated Hospital of Xi’an Jiaotong University from 2009 to 2015. The results indicated that PLK2 was expressed predominantly in the cytoplasm cytomembrane of low-grade glioma and non-tumor samples, however, the staining was significantly lower in GBM samples (Supplementary Fig. 1A). Kaplan-Meier survival analysis showed that glioma patients with lower expression of PLK2 represented a shorter overall survival when compared with higher PLK2 expression (Fig. 2d). To confirm these findings, 676 patients’ samples from Rembrandt database were analyzed and similar results were observed (Fig. 2e). Although underwent post-surgery radiotherapy or TMZ chemotherapy, the overall survival was unfavorable in PLK2 low expressed patients (Supplementary Fig. 1D and E). Furthermore, the glioma patients were divided into 2 groups according to the GIS of PLK2 expression. The results indicated that PLK2 was likely to be enriched in low grade gliomas compared with GBM (Supplementary Fig. 1B). Taken together, these data demonstrated that knock-down of PLK2 is associated with pathological malignancy and revealed severe outcomes in glioma.

### PLK2 overexpression reduces the malignancy of GBM cells both in vitro and in vivo

**Fig. 3:**
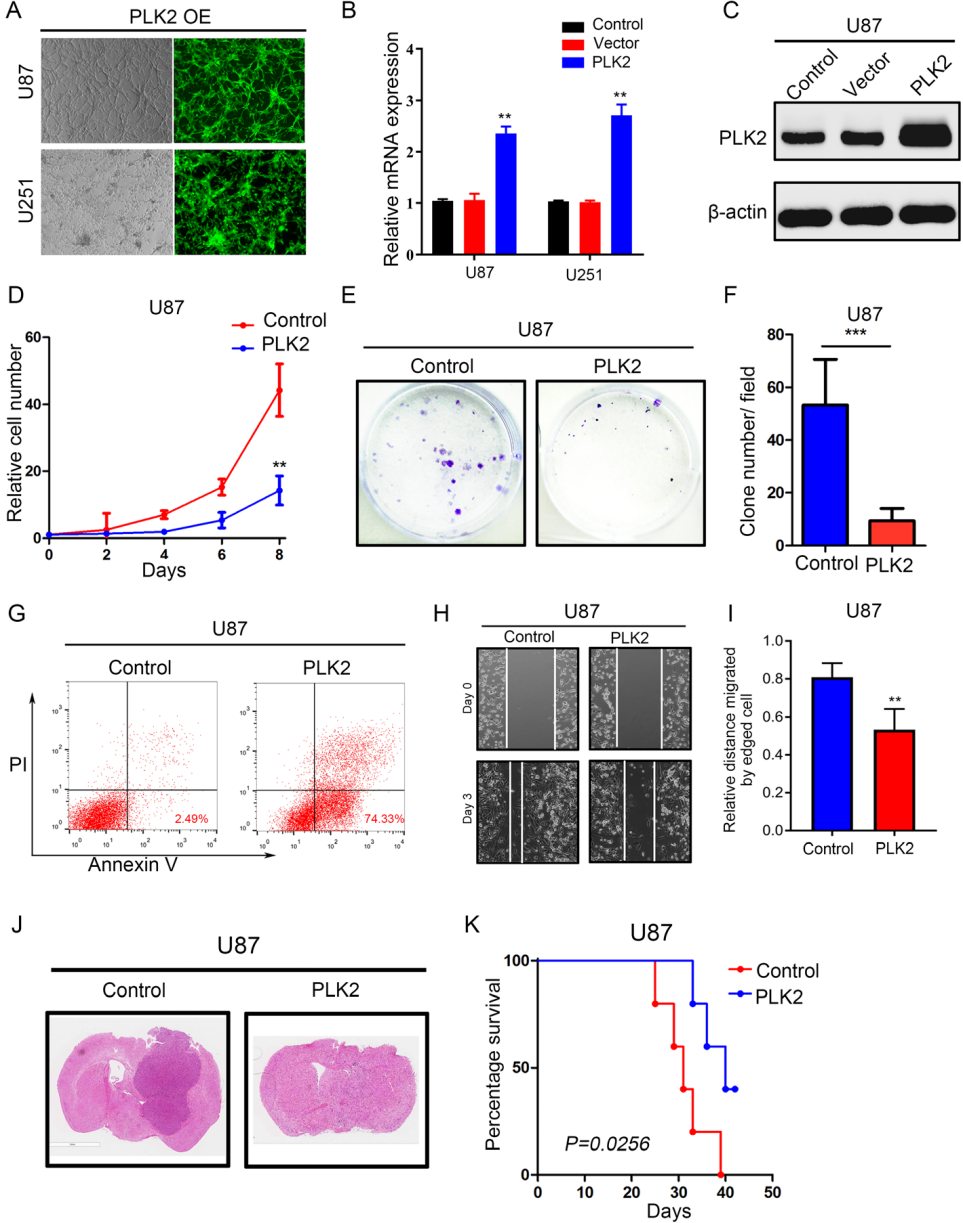
PLK2 overexpression reduces the malignancy of GBM cells both in vitro and in vivo*.* **a**, Representative images of immunofluorescence showing the transduction efficiency of U87 and U251 cell lines after lentiviral PLK2 transduction. **b**, qRT-PCR analysis for measuring the mRNA expression of PLK2 in U87 and U251 cells after lentiviral PLK2 transduction (**P < .001, with student’s t-test). **c**, Western blot analysis for detecting the PLK2 protein expression in U87 glioma cell line transduced with lentiviral PLK2, lentiviral vector and blank control. β-actin was used as an internal control. **d**, Time survival curve of U87 cell line transduced with lentiviral PLK2 and negative control (***P < .001, with one-way ANOVA followed by Dunnett’s post-test). **e** and **f**, the colony formation ability of U87 cells pre-treated with either lentiviral vector or PLK2 (***P < .001, with student t-test). **g**, Flow cytometry analyses using Annexin V and Propidium Iodide for apoptotic ratio analysis in U87 cells pretreated with indicated interventions. **h** and **i** the migratory ability of U87 cells pre-treated with either lentiviral vector or PLK2. **j**, Representative images of H&E-stained mouse brain sections after the intracranial transplantation of indicated U87 cell lines. Scale bars: 3 mm H, Kaplan-Meier curve comparing the overall survival of xenograft mice with U87 cells pre-treated with either lentiviral vector or PLK2 (*P* = 0.0256, with log-rank test). All data were presented as the mean ± SD of triplicate independent experiments

To further study the function of PLK2, exogenous PLK2 was introduced into U87 and U251 cell lines through lentiviral transduction. The transduction efficiency was confirmed by immunofluorescence imaging (Fig. 3a). qRT-PCR (Fig. 3b) and western blot assays (Fig. 3c; Supplementary Fig. 2A) showed that PLK2 expression was significantly increased in both U87 and U251 cells transduced with PLK2 overexpression lentivirus compared with negative control. To explore the effect of PLK2 overexpression on tumor malignancy in GBM cells, in vitro cell proliferation assays as well as colony formation assays were performed. The results indicated that PLK2 overexpression impaired cell growth and self-renewal ability (Fig. 3d-f, Supplementary Fig. 2B-D). Moreover, the apoptotic rate of glioma cells transduced with PLK2 overexpression lentivirus increased significantly compared with its negative control (Fig. 3g, Supplementary Fig. 2E). According to the cell migration assays, the migratory ability of U87 and U251 cell lines were significantly decreased by PLK2 overexpression (Fig. 3h and i, Supplementary Fig. 2F and G). Additionally, the intracranial xenograft mouse model was constructed to investigate the function of PLK2 on tumorigenesis. As a result, most of the mice transplanted with PLK2 overexpressed U87 cell line failed to form tumors (Fig. 3j). Kaplan-Meier analysis showed elevated PLK2 suppressed the in vivo tumor growth thus prolonged the survival of xenograft mice (Fig. 3j, Supplementary Fig. 2H). Altogether, these data showed that PLK2 overexpression reduced the malignancy including proliferation, migration, self-renewal, and tumorigenesis of GBM cells both in vitro and in vivo.

### Elevated PLK2 promotes chemosensitivity in GBM

**Fig. 4:**
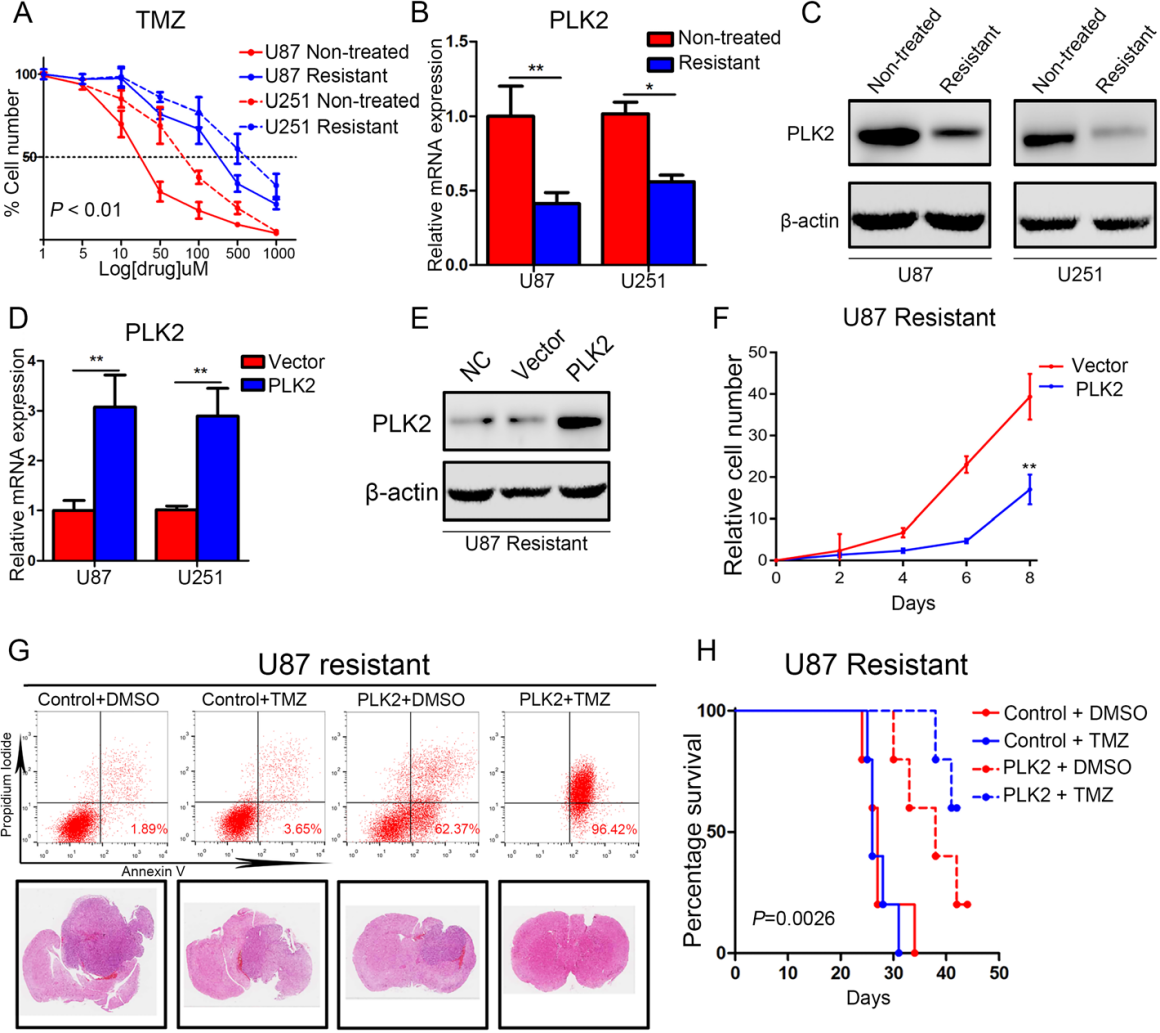
Elevated PLK2 promotes chemosensitivity in GBM. **a**, Concentration survival curve of U87 and U251 TMZ-resistant cell lines and non-treated cell lines (*P* < 0.01, with one-way ANOVA followed by Dunnett’s post-test). **b**, qRT-PCR analysis for measuring the mRNA expression of PLK2 in glioma TMZ-resistant cell lines versus non-treated cell lines. **c**, Western blot assays to compare the PLK2 protein expression in TMZ resistant glioma cell lines with non-treated cell lines. β-actin was used as an internal control. **d**, qRT-PCR assays to measure the mRNA expression level of PLK2 in TMZ resistant cell lines when transduced with lentiviral PLK2 and negative control (**P < 0.01, with student’s t-test). **e**, Western blot analysis to detect the overexpression efficiency of lentiviral PLK2 in U87 resistant cell liens. β-actin was used as an internal control. **f**, In vitro cell proliferation assays were performed by using different interventions as indicated in U87 cell line. (**P < .01, with one-way ANOVA followed by Dunnett’s post-test). **g**, Upper panel: Flow cytometry analyses using Annexin V and Propidium Iodide for apoptotic ratio analysis in U87 resistant cells pretreated with indicated interventions. Lower panel: Representative images of H&E-stained mouse brain sections after the intracranial transplantation with indicated U87 cell lines. **h**, Kaplan-Meier analysis for in vivo intracranial xenograft mice using U87 cells pre-transduced with PLK2 lentivirus and negative control (*P* = 0.0026, with log-rank test). All data were presented as the mean ± SD of triplicate independent experiments

To further investigate the correlation between PLK2 and acquired TMZ resistance, TMZ resistant U87 and U251 cell lines were constructed before cell viability assays. As is shown in Fig. 4a, resistant cell lines showed less vulnerability to TMZ treatment compared with non-treated naïve cell lines. Additionally, down-regulated PLK2 expression was found in both U87 and U251 resistant cell lines (Fig. 4b, c). We then introduced PLK2 overexpression lentivirus into TMZ resistant cell lines and measure the transduction efficiency by immunofluorescence (Supplementary Fig. 3A). qRT-PCR analysis illustrated that the PLK2 mRNA expression was significantly increased in both U87 and U251 transfected with PLK2 overexpression lentivirus compared with the control cells (Fig. 4d). Consistently, the protein levels of PLK2 were also up-regulated by exogenous lentiviral overexpression (Fig. 4e). In vitro cell proliferation assays were then performed to investigate the function of PLK2 overexpression on acquired TMZ resistance. The results showed that enhanced PLK2 expression significantly attenuated the proliferation of resistant U87 and U251 cell lines (Fig. 4f, Supplementary Fig. 3B). Furthermore, the flowcytometry assays were used to investigate the apoptosis of U87 and U251 resistant cell lines when treated with a combination of TMZ and PLK2 overexpression. Notably, PLK2 overexpression significantly elevated the apoptosis rate of resistant glioma cell lines under the treatment of TMZ (Fig. 4g, Supplementary Fig. 3C). Also, the in vivo xenograft mouse model was constructed using TMZ resistant U87 and U251 cell lines, and mice transplanted with PLK2 overexpressed U87 resistant cell lines became more sensitive to TMZ treatment compared with the control groups. Consistently, Kaplan-Meier analysis showed prolonged survival time in PLK2 overexpression mice group when treated with TMZ (Fig. 4h, Supplementary Fig. 3D). Taken together, these findings indicated that loss of PLK2 promotes acquired TMZ resistance of GBM while increased PLK2 expression enhances TMZ-sensitivity of GBM.

### Loss of PLK2 enhances TMZ resistance of GBM via activation of notch signaling

**Fig. 5:**
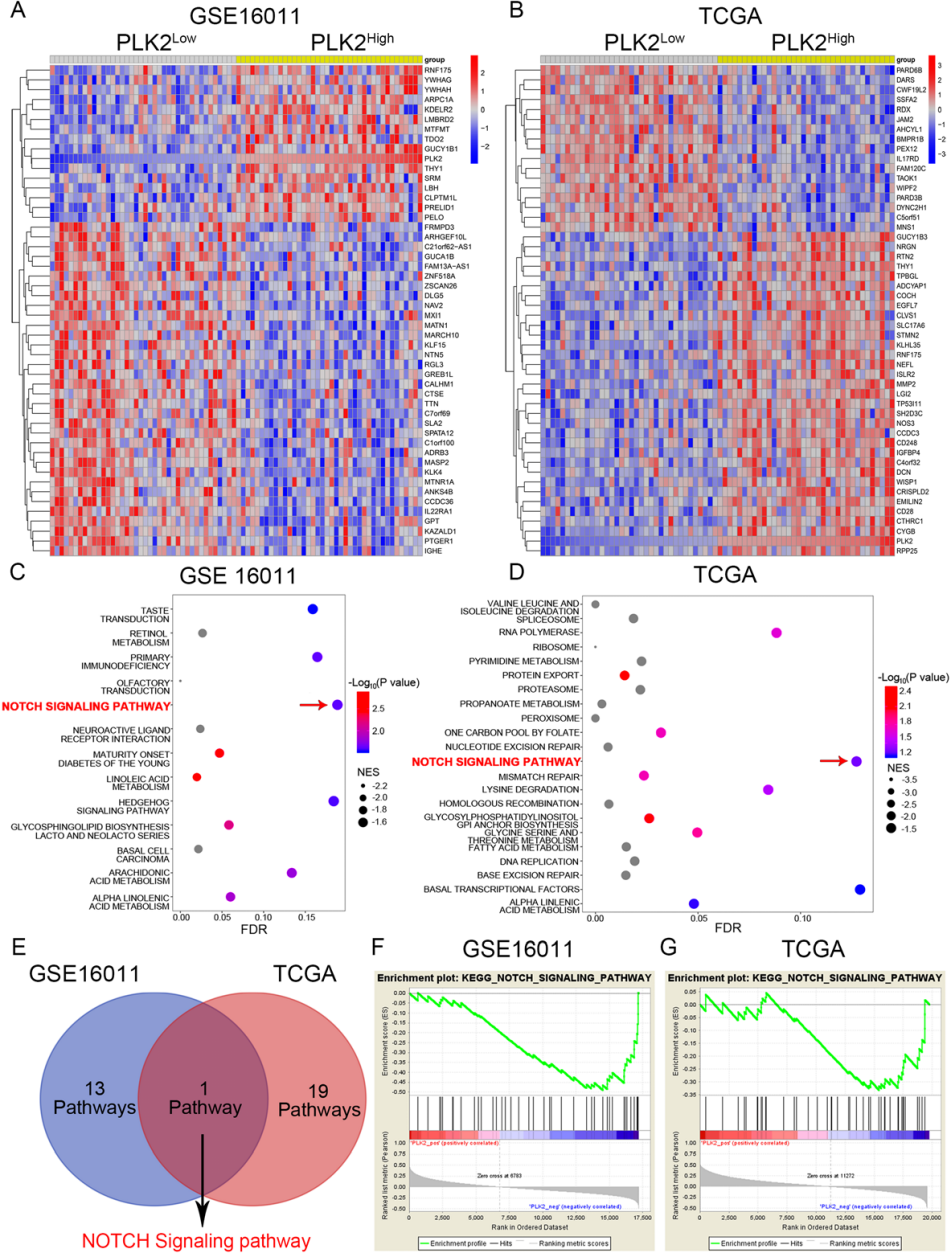
Loss of PLK2 enhances TMZ resistance of GBM via activation of Notch signaling. **a** and **b**, Hierarchical bi-clustering analysis was performed by using GEO database (GSE16011) or TCGA GBM database, indicating the significant gene signature in PLK2^high^ GBM compared with PLK2^low^ GBM. **c** and **d**, Bubble plots were carried out to illustrate the results of GSEA analyses using the transcriptome profiles of GSE16011 and TCGA GBM, indicating that PLK2 was negatively correlated with these pathways. **e**, Venn diagram of all significantly enriched pathways that are negatively correlated with PLK2 from GSEA analyses, showing Notch signaling pathway was the only common pathway that is negatively correlated with PLK2. **f**, GSEA plot showed the negative correlation between PLK2 and Notch signaling pathway

As we previously demonstrated that PLK2 functions as a tumor-suppressor gene and attenuated chemoresistance in GBM, it is essential to deeply investigate the underlying signaling pathways regulated by PLK2. To this end, bioinformatics analysis with TCGA dataset and GSE16011 dataset were performed. The transcriptome profiles of GSE16011 and TCGA datasets were respectively grouped into 2 groups according to the expression of PLK2. Hierarchical bi-clustering analysis indicated significant gene signatures in PLK2^high^ and PLK2^low^ GBM (Fig. 5a, b). GSEA analysis were then performed to figure out the negatively correlated pathways of PLK2. As is shown in Fig. 5c-f, Notch signaling pathway was the only pathway that was simultaneously enriched in PLK2^low^ group in both GSE16011 and TCGA databases. Therefore, we choose Notch signaling pathway as a candidate downstream pathway of PLK2.

**Fig. 6:**
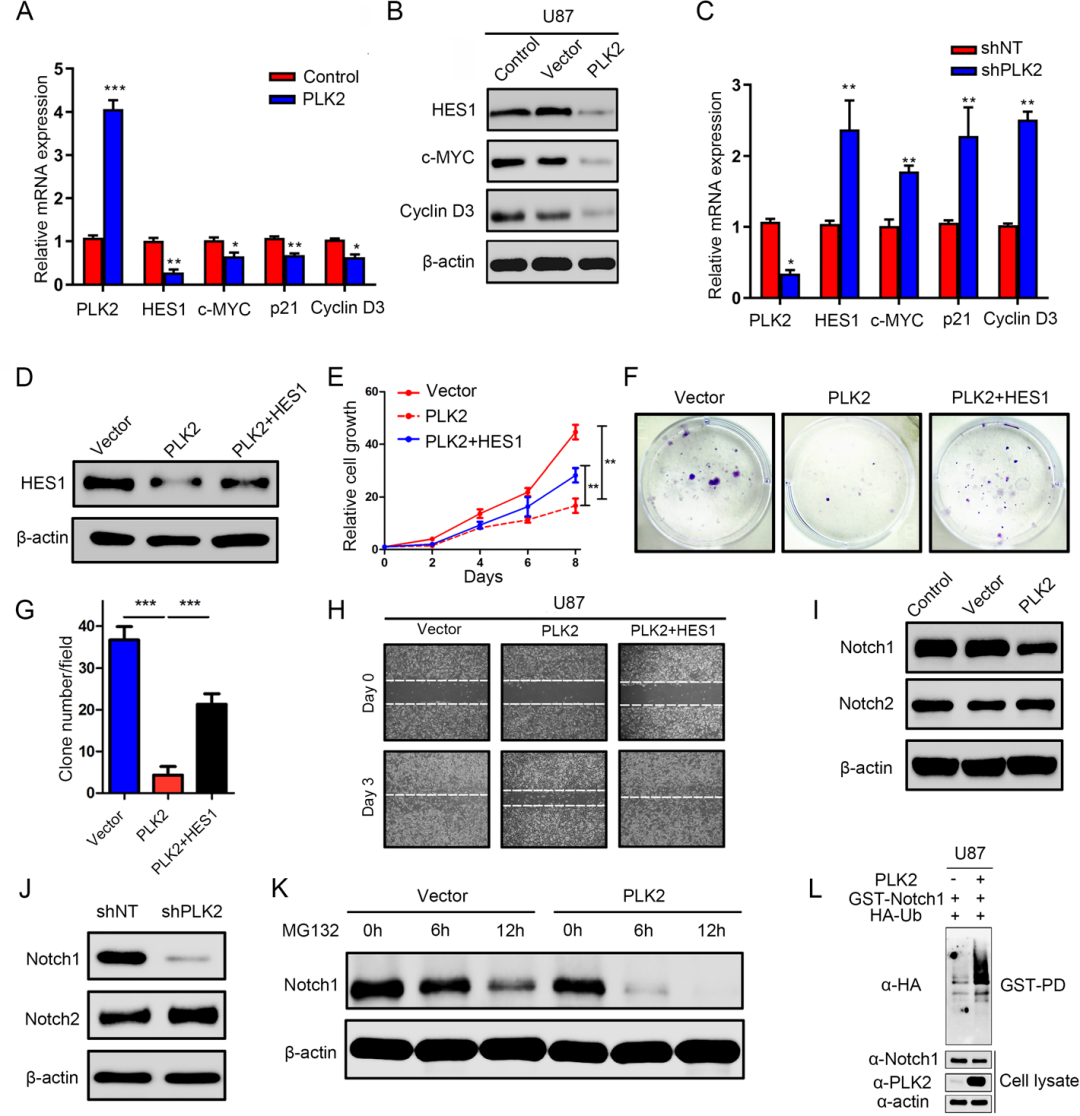
Loss of PLK2 enhances TMZ resistance of GBM via activation of Notch signaling. **a**, qRT-PCR assays were performed to measure the mRNA expression levels of the downstream targets of Notch signaling when cells were pre-transduced with lentiviral PLK2 and its negative control. **b**, Western blot analyses were conducted to detect the protein levels of downstream targets of Notch signaling. β-actin was used as an internal control. **c**, qRT-PCR assays were carried out to measure the mRNA expression levels of Notch downstream targets when treated with lentiviral shPLK2. **d**, Western blot assays was used to detect the protein level of HES1 in PLK2 OE U87 cells when transduced with HES1 lentivirus. **e**, Time survival curve of U87 cell pretreated with indicating interventions. **f** and **g**, Colony formation ability of U87 cells pretreated with different lentiviruses as indicated ((***P < .001, with student’s t-test). **h**, representative images of wound healing assays to indicate the migratory ability of U87 cells pretreated with different lentiviruses. **i**, the effect of PLK2 OE on activated Notch1 and Notch2 protein was detect by western blot, β-actin was used as an internal control. **j**, the effect of PLK2 knockdown on activated Notch1 and Notch2 protein was detected by western blot, β-actin was used as an internal control. **k**, the effect of proteasome inhibitor MG132 on protein level of Notch1 pretreated with or without PLK2 OE lentivirus, β-actin was used as an internal control. **l**, GST-pulldown assay showed that the ubiquitination level of GST-Notch1 increased when PLK2 was overexpressed compare with control group in U87 cell line. All data were presented as the mean ± SD of triplicate independent experiments

To further confirm the results of the potential mechanism underlying PLK2 and Notch, qRT-PCR and Western blot assays were then carried out. The results showed that PLK2 overexpression significantly reduced the downstream targets of Notch signaling pathway including HES1, cMYC, p21, Cyclin D3 on transcriptome and translation levels (Fig. 6a, b, Supplementary Fig. 4A, B). Additionally, PLK2 knock-down considerably enhanced these responding targets of Notch signaling pathways (Fig. 6c, Supplementary Fig. 4C). To further investigate the potential mechanism by which PLK2 overexpression suppresses Notch signaling pathway, we overexpressed HES1 in lentiviral PLK2 overexpressed U87 cells (Fig. 6d, Supplementary Fig. 4D). Notably, the suppression of cell growth (Fig. 6e), colony formation (Fig. 6f, g, Supplementary Fig. 4F, G) and migratory ability (Fig. 6h, Supplementary Fig. 4H) by PLK2 overexpression can be partially rescued when combined with HES1 overexpression, indicating that PLK2 reduces the malignancy of GBM cells by inactivation of Notch signaling pathway via transcriptional regulation of HES1.

To further elucidate the effect of PLK2 on Notch signaling activation, Western blot analyses were performed to investigate the expression of activated Notch1 and Notch2 in U87 cell line. The result demonstrated that Notch 1 expression was remarkably reduced after exogenous overexpression of PLK2, however, the expression of Notch2 was not significantly affected (Fig. 6i, Supplementary Fig. 4I). Consistently, shRNA-induced suppression of PLK2 increased Notch1 protein in GBM, indicating that Notch1 was an essential regulator through which PLK2 could inactivate Notch pathway (Fig. 6j, Supplementary Fig. 4 J). To validate this, protein stability assays were performed with U87 cells transduced with or without PLK2 overexpression lentivirus by using MG132, which was used to block the protein proteolysis. The results showed that protein stability of Notch1 was significantly reduced after PLK2 overexpression in U87 cell (Fig. 6k, Supplementary Fig. 4 K). Consistent with these results, ubiquitinated Notch levels were clearly increased in PLK2 overexpressed samples compared with levels in control groups (Fig. 6l and Supplementary Fig. 4 L), indicating that loss of PLK2 stabilized Notch1 through suppressing degradation of Notch1 protein then activated Notch signaling, which finally leaded to acquired TMZ resistance and tumor recurrence.

## Discussion

Most targeted anti-GBM treatments including cytotoxic and radiation therapies are encumbered by the development of resistance in GBM cells [38]. The limited effectiveness of TMZ in GBM has been correlated with its inability to induce adequate level of apoptosis thus leads to acquired chemoresistance [39]. Notably, resistance is a complicated process involving deficiencies in apoptosis induction, activation of multiple signaling pathways and expulsion of drug through cell membrane transporters [38, 40]. Although novel therapeutic targets have been found in the past several decades, the overall survival of GBM patients remain dismal due to the tumor recurrence followed by chemoresistance [3, 41]. Therefore, developing new biomarkers and therapeutic strategies targeting TMZ resistance in GBM are in urgent need.

Increasing evidence has identified that the aberrant expression of tumor-related kinases is closely connected to the progression, therapeutic resistance or recurrence of various cancers [42–45]. The polo-like kinases (PLKs) are among these protein kinases that control multiple complex process of centriole duplication [46]. The improvement of the basic biology of PLK1 have helped us to deeply understand its function in regulating the cell cycle [47], and various observations on the upregulation of PLK1 in human cancers also provided researchers a new anti-tumor target [48, 49]. For instance, the inhibition of PLK1 was reported to cause cell cycle arrest and lead to cell death in glioblastoma [50]. Unlike PLK1, PLK2 functions as a tumor suppressor in some cancer types. In a wide range of B-cell neoplasms, PLK2 was epigenetically silenced due to the aberrant CpG methylation [13]. However, recent bioinformatic approaches demonstrated that hypermethylation of PLK2 might lead to a favorable outcome in GBM patients, which need to be further confirmed by experimental studies [51]. Additionally, the ectopic expression of PLK2 in Burkitt lymphoma cells increased the apoptosis rate, indicating the tumor-suppressing function of PLK2 in human cancers [13]. Moreover, another indirect evidence for this tumor suppressor was discovered in a study of acute myeloma. This study demonstrated that targeting PLK2 by miR-126 inhibits cell apoptosis and increases cell viability, indicating the silencing of PLK2 enhances the cell viability in acute myeloma cell lines [14]. However, the functional role of PLK2 in GBM chemoresistance has not been fully elucidated. In this study, we demonstrated that PLK2 is down-regulated in TMZ resistant GBM cells and it is negatively associated with clinical outcome. Furthermore, we demonstrated that PLK2 overexpression attenuated TMZ resistance in GBM cell lines by destabilizing NOTCH1.

The cleavage of notch receptor by γ-secretase after binding of ligands like jagged or delta family leads to the translocation of Notch intracellular domain (NICD) to the nucleus, thereby activating notch-related downstream targets [52]. Afterwards, notch signaling is terminated by triggering ubiquitinated degradation of NICD PEST domain with the absence of SCF E3 ubiquitin ligase [53, 54]. And the recognition of NICD by this ligase is mainly mediated by FBXW7, which binds to the phosphorylated NICD to recruit E3 ubiquitin ligase [55]. Mutations within the PEST domain of NICD or mutation of FBXW7 result in NICD stabilization, thereby leading to abnormal activation of its downstream targets [54–56]. Accumulating evidence shows the phosphorylation of NICD enhances FBXW7 binding, indicating a potential mechanism to suppress notch signaling by NICD ubiquitination [54]. Moreover, Fryer et al. [57] clarified inhibition of CDKs by small molecule inhibitors results in the stabilization of NICD, leading to higher NICD levels and half-life, thereby slowing down oscillations in the segmentation clock. These previous studies indicated that the activation or maintenance of notch signaling are partially controlled by the interaction between kinases and NICD. Although the Notch signaling has been clarified to be involved in the progression of various cancers, the correlation of Notch1 and PLK2 has not been investigated. Therefore, we hypothesize that PLK2 may act as a suppressor of notch signaling by phosphorylating NICD. To this end, we demonstrated that PLK2 suppressed Notch signaling pathway by destabilizing activated Notch1 thus lead to the down-regulation of its downstream targets including HES1. Moreover, the simultaneous overexpression of PLK2 and HES1 partially rescued the suppression of PLK2 on Notch signaling pathway and its biological functions. In addition, the degradation of activated Notch1 was observed when PLK2 was overexpressed, indicating that PLK2 might phosphorylate the Notch1 thus lead to the ubiquitination of activated Notch1. However, further experiments are needed to investigate the mechanism of how PLK2 phosphorylate Notch1.

It has been widely proved that Notch signaling is at the center of a signaling network, which also includes pathways such as PI3K/AKT, NF-κB, STAT3, Hedgehog, and Wnt/β-catenin, which are involved in cell differentiation, cell growth, and survival. Therefore, targeting Notch signaling might be a potential therapeutic target for GBM particularly for those which are resistant to TMZ. Although PLK2-Notch-targeted monotherapy seems to be promising, a better accomplishment is to combine Notch inhibitors with other molecules or chemotherapeutic agents like bevacizumab and TMZ, respectively. Additionally, Meng et al. [58] found the simultaneous inhibition of EGFR and MET signaling by a novel nanoparticle enhanced TMZ chemosensitivity in TMZ-resistant cell lines, indicating the significance of targeting different pathways that contribute to chemoresistance. Although the underlying roles of PLK2 in chemoresistance are well discussed in this study, additional molecular biological investigations are still required for evaluating the clinical significance of PLK2 in chemotherapy. Notably, although the samples from our center indicated that there might be a correlation between PLK2 expression and clinical degree of glioma, data from other databases including TCGA, CGGA, Rembrandt indicated an ambiguous correlation between PLK2 and tumor grade or cancer pathological subtypes. This may be due to the small scale of our samples and no conclusion related to the correlation between WHO grades and PLK2 expression could be made from this finding. Moreover, integrated multi-central data and meta analyses are needed to further confirm this finding. Also, how PLK2 interacts with NOTCH1 to further regulate notch signaling and whether PLK2-induced chemosensitivity solely depends on its regulation on notch signaling remain unclear. Further investigations like GST pull-down and immunoprecipitation assays are needed to study the ubiquitination of Notch1 caused by PLK2 overexpression. Moreover, alteration of a single candidate biomarker might bring unpredictable effects due the heterogeneity of GBM. Therefore, it is crucial to evaluate GBM patients by more integrated strategies before clinical management.

## Conclusion

In this study, we analyzed the transcriptome profiles of different public databases including Gene Expression Omnibus (GEO) and The Cancer Genome Atlas (TCGA) to identify PLK2 as a candidate kinase in regulating TMZ resistance in GBM. Notably, depressed PLK2 expression was found to be strongly related to poor prognosis in GBM patients. Next, experimental analyses were performed to clarify the underlying role of PLK2 by lentiviral transduction, and we demonstrated that PLK2 promoted chemosensitivity of GBM cell lines to TMZ by in vitro biological assays. Moreover, the xenograft mouse model illustrated that PLK2-OE prolonged the overall survival of tumor-bearing mice and consistently increased the chemosensitivity to TMZ. In addition, Notch signaling was the only overlapped signaling pathway which negatively correlated with PLK2 in different databases according to the result of GSEA analyses. We further confirmed this finding by molecular biological assays and demonstrated that PLK2 suppressed Notch signaling pathway and its biological functions by destabilizing activated Notch1.

## Supplementary Information

**Additional file 1: Supplementary Fig. 1.** Decreased PLK2 expression is closely associated with poor outcomes in glioma patients. A, Representative IHC images of PLK2 in low-grade glioma and GBM samples. Upper panel: PLK2 staining; Lower panel: H & E staining. B, PLK2 was silenced in high-grade glioma samples. PLK2^low^ samples accounted for 57.63% of GBM, while in PLK2^high^ glioma samples, GBM accounted for 9.38%. C, Differential gene expression analyses were performed using TCGA Affymetrix HT HG U133A dataset to compare the expression of PLK2 in different subtypes of GBM (***P < .001, with one-way ANOVA followed by Dunnett’s posttest). D, Kaplan-Meier analysis for patients underwent post-surgery radiotherapy (*P* = 0.0002, with log-rank test). E, Kaplan-Meier analysis for patients underwent post-surgery TMZ therapy (*P*-0.0006, with log-rank test).

**Additional file 2: Supplementary Fig. 2.** PLK2 overexpression reduces the malignancy of GBM cell lines in vitro and in vivo*.* A, Western blot analysis for detecting the PLK2 protein expression in U251 glioma cell line transduced with lentiviral PLK2, lentiviral vector and blank control. β-actin was used as an internal control. B, Time survival curve of U251 cell line transduced with lentiviral PLK2 and negative control (***P < .001, with one-way ANOVA followed by Dunnett’s post-test). C and D, the colony formation ability of U251 cells pre-treated with either lentiviral vector or PLK2 (***P < .001, with student t-test). E, Flow cytometry analyses using Annexin V and Propidium Iodide for apoptotic ratio analysis in U251 cells pretreated with indicated interventions. F and G, the migratory ability of U251 cells pre-treated with either lentiviral vector or PLK2. H, Kaplan-Meier curve comparing the overall survival of xenograft mice with U251 cells pre-treated with either lentiviral vector or PLK2 (*P* = 0.0132, with log-rank test). All data were presented as the mean ± SD of triplicate independent experiments.

**Additional file 3: Supplementary Fig. 3.** Elevated PLK2 promotes chemosensitivity in GBM. A, Representative images of immunofluorescence showing the transduction efficiency of U87 and U251 TMZ-resistant cell lines after lentiviral PLK2 transduction. B, In vitro cell proliferation assays were performed by using different interventions as indicated in U251 cell line. (**P < .01, with one-way ANOVA followed by Dunnett’s post-test). C, Flow cytometry analyses using Annexin V and Propidium Iodide for apoptotic ratio analyses of U251 resistant cells pretreated with indicated interventions. D, Kaplan-Meier analysis for in vivo intracranial xenograft mice using U251 cells pre-transduced with PLK2 lentivirus and negative control (*P* = 0.0052, with log-rank test). All data were presented as the mean ± SD of triplicate independent experiments.

**Additional file 4: Supplementary Fig. 4.** Loss of PLK2 enhances TMZ resistance of GBM via activation of Notch signaling. A, qRT-PCR assays were performed to measure the mRNA expression levels of the downstream targets of Notch signaling when cells were pre-transduced with lentiviral PLK2 and its negative control. B, Western blot analyses were conducted to detect the protein levels of downstream targets of Notch signaling. β-actin was used as an internal control. C, qRT-PCR assays were carried out to measure the mRNA expression levels of Notch downstream targets when treated with lentiviral shPLK2. D, Western blot assays was used to detect the protein level of HES1 in PLK2 OE U251 cells when transduced with HES1 lentivirus. E, Time survival curve of U251 cell pretreated with indicating interventions. F and G, Colony formation ability of U251 cells pretreated with different lentiviruses as indicated ((***P < .001, with student’s t-test). H, representative images of wound healing assays to indicate the migratory ability of U251 cells pretreated with different lentiviruses. I, the effect of PLK2 OE on activated Notch1 and Notch2 protein was detect by western blot, β-actin was used as an internal control. J, the effect of PLK2 knockdown on activated Notch1 and Notch2 protein was detected by western blot, β-actin was used as an internal control. K, the effect of proteasome inhibitor MG132 on protein level of Notch1 pretreated with or without PLK2 OE lentivirus, β-actin was used as an internal control. L, GST-pulldown assay showed that the ubiquitination level of GST-Notch1 increased when PLK2 was overexpressed compare with control group in U251 cell line. All data were presented as the mean ± SD of triplicate independent experiments.

**Additional file 5: Supplementary Fig. 5.** Schematic for this study indicating the interaction between PLK2 and notch signaling pathway and its potential downstream effects.

**Additional file 6: Supplementary Table 1.** The differentially expressed kinases in GSE68029.

**Additional file 7: Supplementary Table 2.** The differentially expressed kinases in GSE16011.

**Additional file 8: Supplementary Table 3.** The differentially expressed kinases in TCGA GBM.

## Data Availability

Not applicable.

## Abbreviations

GBM: Glioblastoma
GEO: Gene expression omnibus
TMZ: Temozolomide
PLK2: Polo like kinase 2
NICD: Notch Intracellular Domain
TCGA: The Cancer Genome Atlas
DLL: Delta-like ligand
JAG: Jagged
NF-κB: Nuclear factor kappa-light-chain-enhancer of activated B cells
AKT: Protein kinase B
EGFR: Epidermal growth factor receptor
TP53: Tumor protein p53
HES1: Hairy and enhancer of split-1
IHC: Immunohistochemistry
GIS: Global histochemistry score
qRT-PCR: Quantitative RT-PCR
GSEA: Gene set enrichment analysis
GAPDH: Glyceraldehyde-3-phosphate dehydrogenase
