# Supplementary figures and images for: Loss of PLK2 induces acquired resistance to temozolomide in GBM via activation of notch signaling
Source: J Exp Clin Cancer Res. 2020 Nov 11;39:239. doi: 10.1186/s13046-020-01750-4 (PMC7657349; doi:10.1186/s13046-020-01750-4)

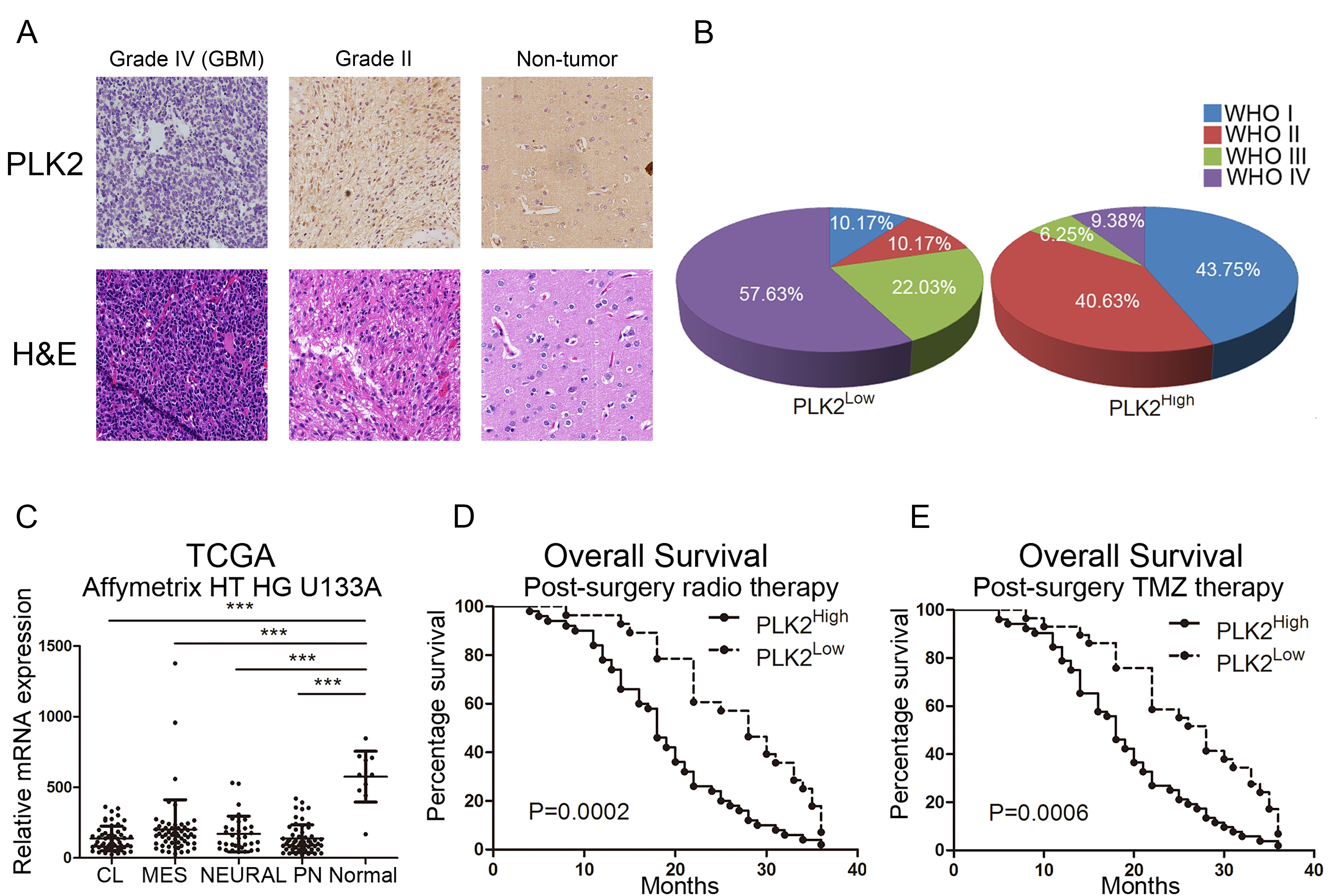

Supplement: Supplementary file 1 — Additional file 1: Supplementary Fig. 1. Decreased PLK2 expression is closely associated with poor outcomes in glioma patients. A, Representative IHC images of PLK2 in low-grade glioma and GBM samples. Upper panel: PLK2 staining; Lower panel: H & E staining. B, PLK2 was silenced in high-grade glioma samples. PLK2low samples accounted for 57.63% of GBM, while in PLK2high glioma samples, GBM accounted for 9.38%. C, Differential gene expression analyses were performed using TCGA Affymetrix HT HG U133A dataset to compare the expression of PLK2 in different subtypes of GBM (***P < .001, with one-way ANOVA followed by Dunnett’s posttest). D, Kaplan-Meier analysis for patients underwent post-surgery radiotherapy (P = 0.0002, with log-rank test). E, Kaplan-Meier analysis for patients underwent post-surgery TMZ therapy (P-0.0006, with log-rank test). [file 13046_2020_1750_MOESM1_ESM.tif]

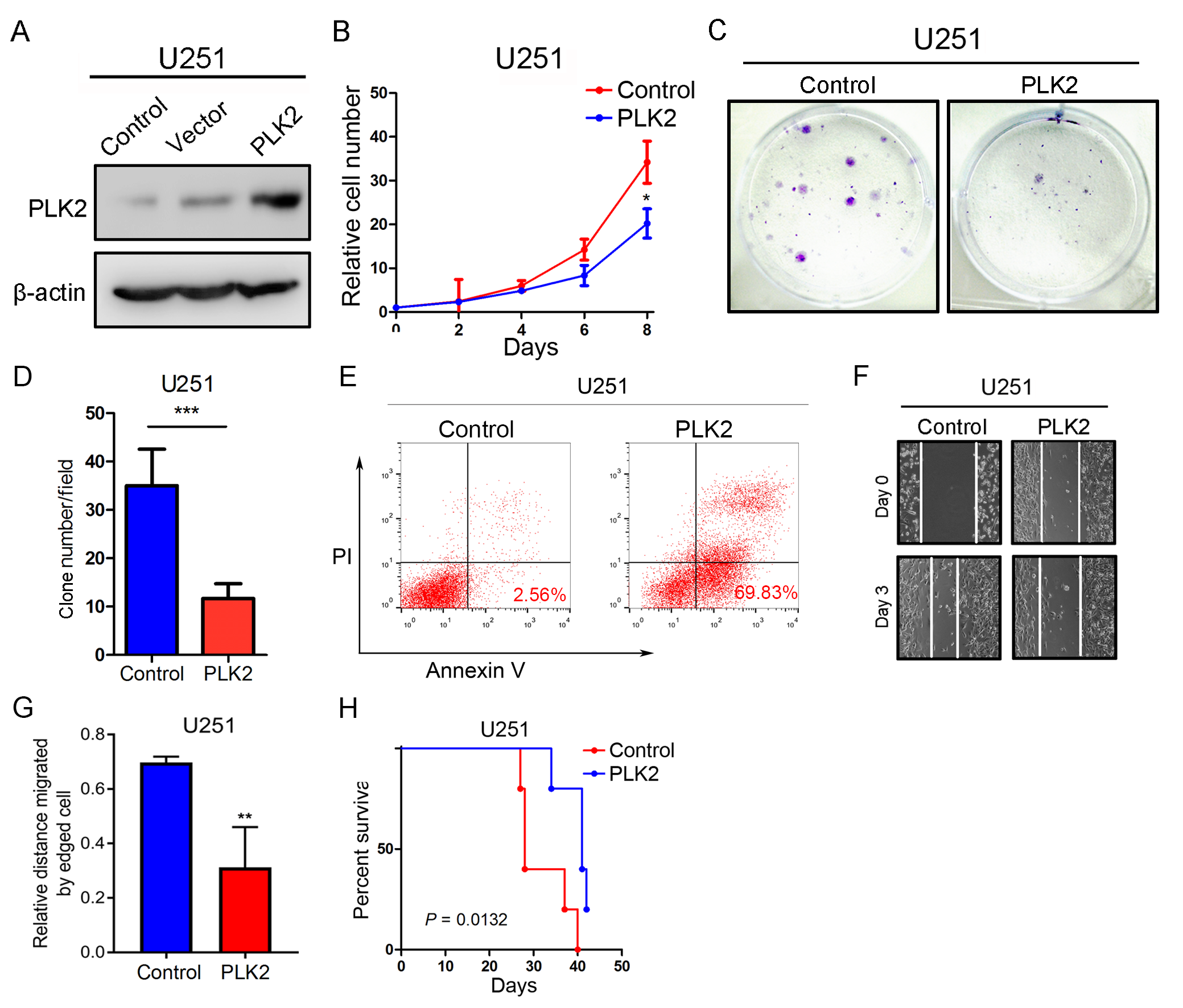

Supplement: Supplementary file 2 — Additional file 2: Supplementary Fig. 2. PLK2 overexpression reduces the malignancy of GBM cell lines in vitro and in vivo. A, Western blot analysis for detecting the PLK2 protein expression in U251 glioma cell line transduced with lentiviral PLK2, lentiviral vector and blank control. β-actin was used as an internal control. B, Time survival curve of U251 cell line transduced with lentiviral PLK2 and negative control (***P < .001, with one-way ANOVA followed by Dunnett’s post-test). C and D, the colony formation ability of U251 cells pre-treated with either lentiviral vector or PLK2 (***P < .001, with student t-test). E, Flow cytometry analyses using Annexin V and Propidium Iodide for apoptotic ratio analysis in U251 cells pretreated with indicated interventions. F and G, the migratory ability of U251 cells pre-treated with either lentiviral vector or PLK2. H, Kaplan-Meier curve comparing the overall survival of xenograft mice with U251 cells pre-treated with either lentiviral vector or PLK2 (P = 0.0132, with log-rank test). All data were presented as the mean ± SD of triplicate independent experiments. [file 13046_2020_1750_MOESM2_ESM.tif]

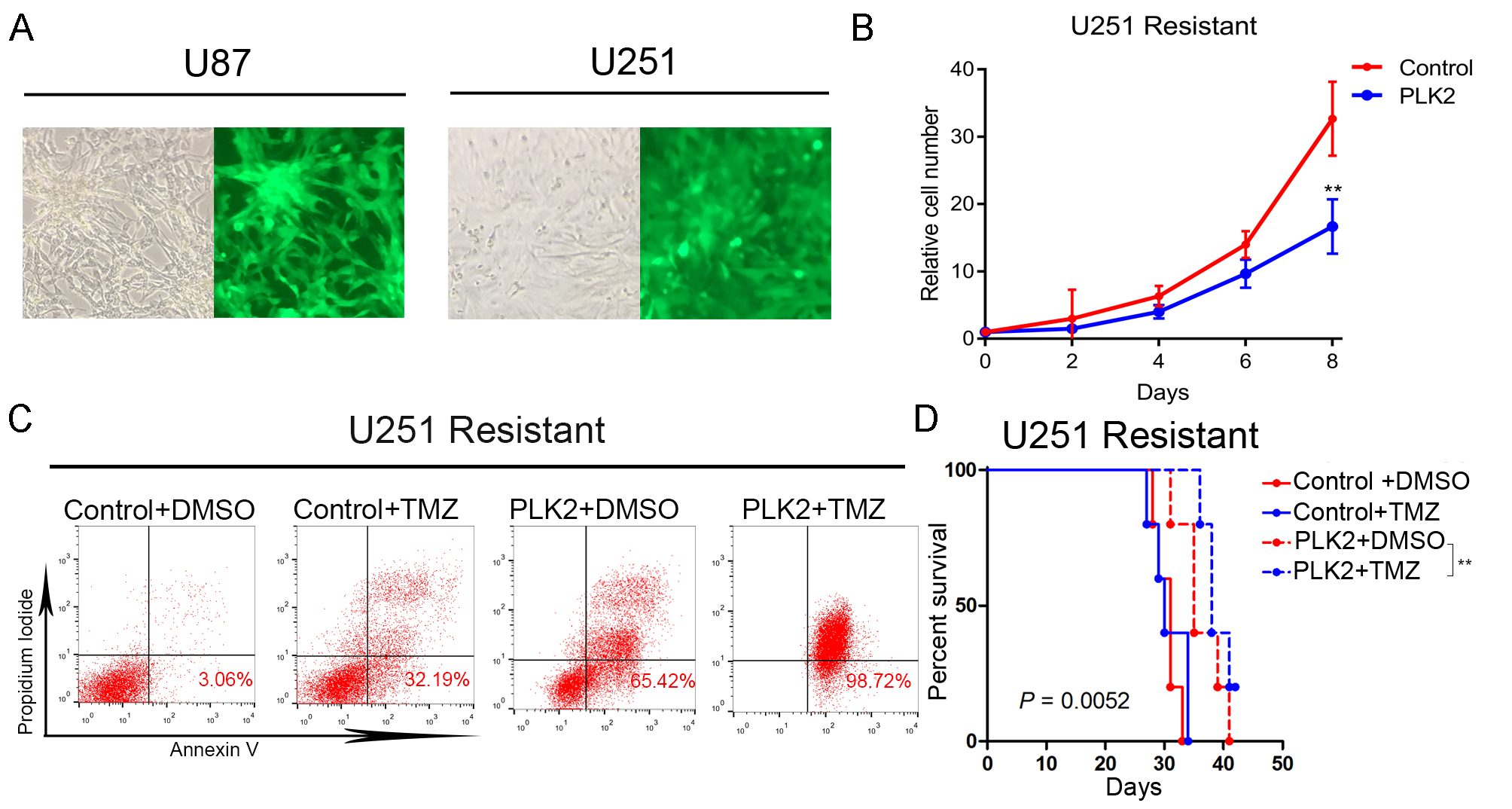

Supplement: Supplementary file 3 — Additional file 3: Supplementary Fig. 3. Elevated PLK2 promotes chemosensitivity in GBM. A, Representative images of immunofluorescence showing the transduction efficiency of U87 and U251 TMZ-resistant cell lines after lentiviral PLK2 transduction. B, In vitro cell proliferation assays were performed by using different interventions as indicated in U251 cell line. (**P < .01, with one-way ANOVA followed by Dunnett’s post-test). C, Flow cytometry analyses using Annexin V and Propidium Iodide for apoptotic ratio analyses of U251 resistant cells pretreated with indicated interventions. D, Kaplan-Meier analysis for in vivo intracranial xenograft mice using U251 cells pre-transduced with PLK2 lentivirus and negative control (P = 0.0052, with log-rank test). All data were presented as the mean ± SD of triplicate independent experiments. [file 13046_2020_1750_MOESM3_ESM.tif]

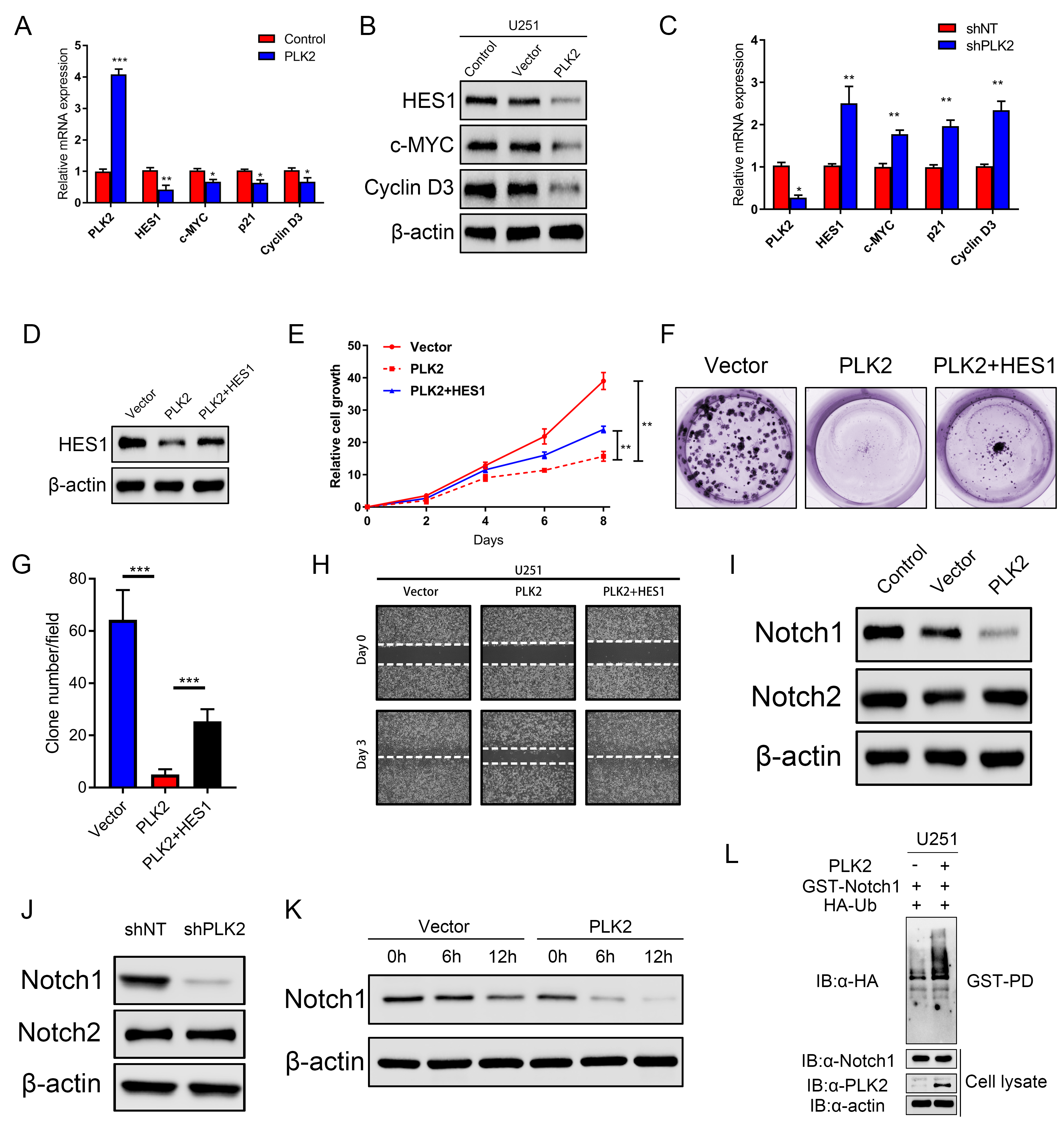

Supplement: Supplementary file 4 — Additional file 4: Supplementary Fig. 4. Loss of PLK2 enhances TMZ resistance of GBM via activation of Notch signaling. A, qRT-PCR assays were performed to measure the mRNA expression levels of the downstream targets of Notch signaling when cells were pre-transduced with lentiviral PLK2 and its negative control. B, Western blot analyses were conducted to detect the protein levels of downstream targets of Notch signaling. β-actin was used as an internal control. C, qRT-PCR assays were carried out to measure the mRNA expression levels of Notch downstream targets when treated with lentiviral shPLK2. D, Western blot assays was used to detect the protein level of HES1 in PLK2 OE U251 cells when transduced with HES1 lentivirus. E, Time survival curve of U251 cell pretreated with indicating interventions. F and G, Colony formation ability of U251 cells pretreated with different lentiviruses as indicated ((***P < .001, with student’s t-test). H, representative images of wound healing assays to indicate the migratory ability of U251 cells pretreated with different lentiviruses. I, the effect of PLK2 OE on activated Notch1 and Notch2 protein was detect by western blot, β-actin was used as an internal control. J, the effect of PLK2 knockdown on activated Notch1 and Notch2 protein was detected by western blot, β-actin was used as an internal control. K, the effect of proteasome inhibitor MG132 on protein level of Notch1 pretreated with or without PLK2 OE lentivirus, β-actin was used as an internal control. L, GST-pulldown assay showed that the ubiquitination level of GST-Notch1 increased when PLK2 was overexpressed compare with control group in U251 cell line. All data were presented as the mean ± SD of triplicate independent experiments. [file 13046_2020_1750_MOESM4_ESM.tif]

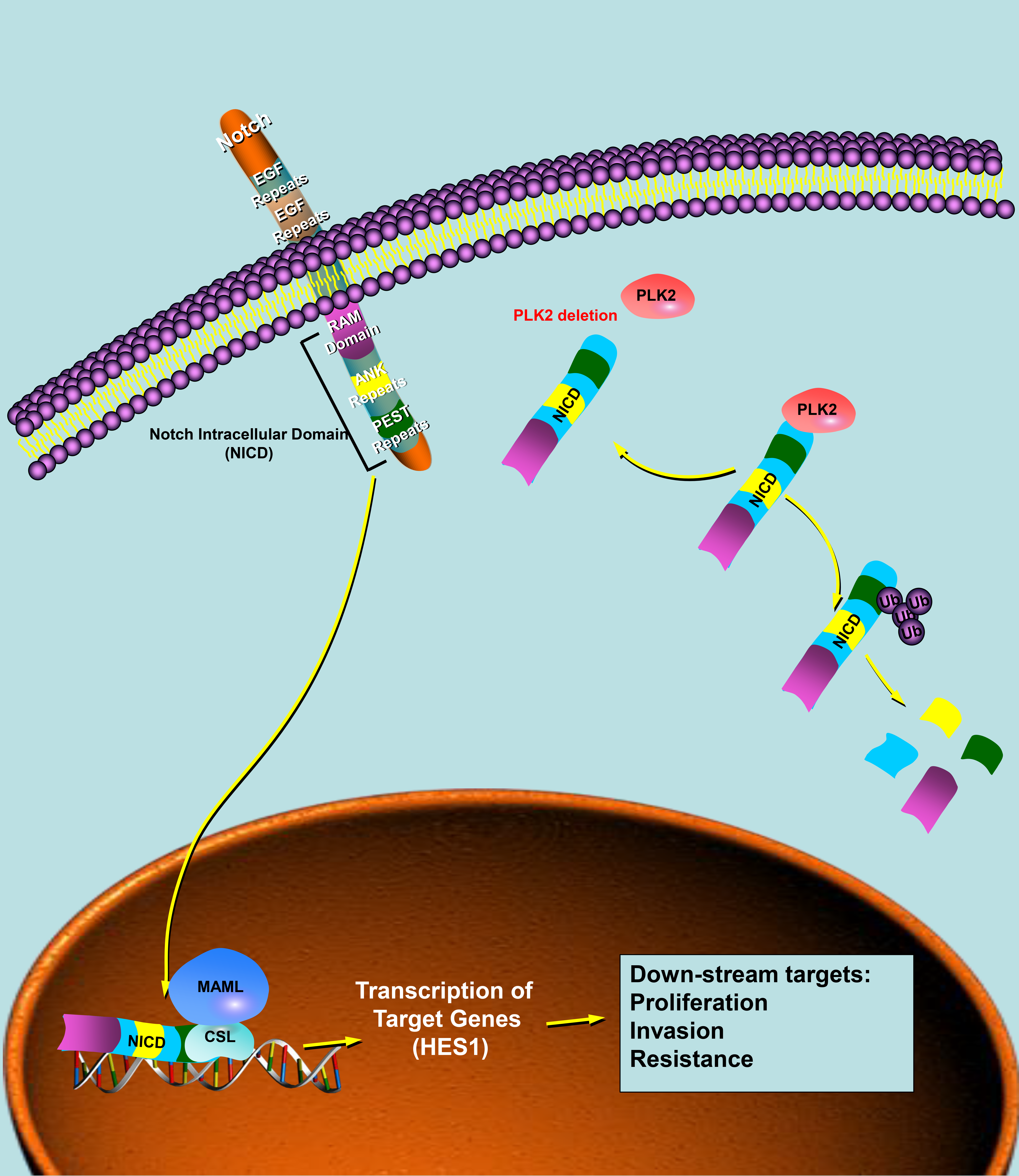

Supplement: Supplementary file 5 — Additional file 5: Supplementary Fig. 5. Schematic for this study indicating the interaction between PLK2 and notch signaling pathway and its potential downstream effects. [file 13046_2020_1750_MOESM5_ESM.tif]
